# Expression of heat shock protein genes in Simmental cattle exposed to heat stress

**DOI:** 10.5713/ab.22.0266

**Published:** 2023-01-11

**Authors:** Luis Felipe Guzmán, Guillermo Martínez-Velázquez, Fernando Villaseñor-González, Vicente Eliezer Vega-Murillo, José Antonio Palacios-Fránquez, Ángel Ríos-Utrera, Moisés Montaño-Bermúdez

**Affiliations:** 1 National Genetic Resources Center, National Institute of Forestry, Agricultural and Livestock Researches, Tepatitlán, Jalisco 47600, Mexico; 2 C.E. Santiago Ixcuintla, National Institute of Forestry, Agricultural and Livestock Researches, Santiago Ixcuintla, Nayarit 63600, Mexico; 3 C.E. Centro Altos de Jalisco, National Institute of Forestry, Agricultural and Livestock Researches, Tepatitlán, Jalisco 47600, Mexico; 4 Faculty of Veterinary Medicine and Zootechnics, Universidad Veracruzana, Veracruz, Veracruz 91710, Mexico; 5 C.E. La Posta, National Institute of Forestry, Agricultural and Livestock Researches, Medellín, Veracruz 94277, Mexico; 6 National Disciplinary Research Center for Physiology and Animal Improvement, National Institute of Forestry, Agricultural and Livestock Researches, Ajuchitán, Queretaro 76280, Mexico

**Keywords:** Heat Stress, *HSP* Family, Gene Expression, Simmental Cattle

## Abstract

**Objective:**

In tropical, subtropical and arid zones, heat stress is the main cause of productivity reduction in cattle. When climate stressors occur, animals become thermal adapted through differential expression of some genes, including heat shock proteins (*HSP*) family. The aim of this study was to determine levels of expression of *HSP60*, *HSP70*, and *HSP90* genes in Simmental cattle raised in tropical environments of Mexico.

**Methods:**

In this study, expression of *HSP60*, *HSP70*, and *HSP90* genes was analyzed in 116 Simmental cattle from three farms with tropical climate located in western Mexico. Animals were sampled twice a day, in the morning and noon. Gene expression was evaluated by quantitative polymerase chain reaction using probes marked with fluorescence. The MIXED procedure of SAS with repeated measures was used for all statistical analysis.

**Results:**

*HSP60* gene expression differences were found for sex (p = 0.0349). *HSP70* gene differences were detected for sampling hour (p = 0.0042), farm (p<0.0001), sex (p = 0.0476), and the interaction sampling hour×farm (p = 0.0002). Gene expression differences for *HSP90* were observed for farm (p<0.0001) and year (p = 0.0521). *HSP70* gene showed to be a better marker of heat stress than *HSP60* and *HSP90* genes.

**Conclusion:**

Expression of *HSP70* gene in Simmental herds of the tropical region of western México was different during early morning and noon, but the expression of the *HSP60* and *HSP90* genes was similar. Identification of resilient animals to heat stress will be useful in the genetic improvement of the Simmental breed.

## INTRODUCTION

Climate change increases environmental temperature impacting livestock industry [1,2]. Climate change affects production systems by modifying variables such temperature, number of warm days and rainfall, among others. Changes in these variables directly and indirectly affect grazing-based livestock production systems, which includes beef production systems. In beef production systems, increases in temperature and in number of warm days per year are associated with heat stress in animals [3]. Heat stress causes a reduction of food intake, a reduction of reproduction and increases animal mortality [1,4].

The Simmental breed has been used in beef production systems in tropical environments as purebred [5], and as part of crossbreeding strategies to develop Simbrah as a composite population. Composite populations are a crossbreeding strategy proposed to counteract heat stress in cattle of tropical regions [6].

Tolerance to heat stress can be quantified through the concentration at the cellular level of a family of proteins called heat shock proteins (HSP). Among the HSP protein family, the HSP70 proteins have the most sensitive response to changes in environmental temperature [7]. In cattle the relationship between gene expression and heat stress have been studied for the *HSP60*, *HSP70*, and *HSP90* genes in *Bos taurus indicus* [8–12] and *Bos taurus taurus* [5,13]. Despite to the progress of research in this topic, only one report in Simmental cattle has been published on the regulation of expression of *HSP70* gene but, none on the regulation of expression of *HSP60* and *HSP90* genes under heat stress.

The aim of this study was to determine levels of expression of *HSP60*, *HSP70*, and *HSP90* genes in Simmental cattle raised in tropical environments of Mexico.

## MATERIALS AND METHODS

### Locations

Between 2018 and 2019, Simmental cattle were sampled from three farms located in the tropical region of western Mexico. Samples were collected at Tangancícuaro in Michoacán State, Puerto Vallarta in Jalisco State, and Compostela in Nayarit State. Environmental temperature (T) and relative humidity (RH) were measured using weather stations installed in each farm. Temperature-humidity index (THI) was estimated with the following equation:


THI=[0.8×T (°C)+(RH [%]/100)×(T [°C]-14.4)+46.4][
14].

Certified veterinarians by the Mexican government obtained the samples. Owners of sampled individuals gave consent for the use of data for research purposes.

### Procedures

One hundred and sixteen Simmental cattle were included in this study, of which, 60, 35, and 21 were from Tangancícuaro, Puerto Vallarta and Compostela, respectively. The ages of the animals ranged from nine months to six years (103 females and 13 males). The study was carried out from June to August of 2018 and 2019, when highest temperatures occurs in these States. Samples were collected in each animal twice a day, in the early morning from 04:00 to 06:00 h, when the temperature is lower, and at noon from 13:00 to 15:00 h, when the temperature is the highest of the day. Cows and calves grazed mostly on Llanero grass (Andropogon gayanus) and Mombaza grass (*Panicum maximum* cv. Mombasa) and were supplemented with a 2.5% molasses-urea mix (1 kg/head/d) during the months of March, April, and May. All animals had *ad libitum* access to minerals (40% salt; 56% calcium orthophosphate; 4% trace minerals) all year round.

A volume of 5 mL of blood were collected from the coccygeal vein in tubes with ethylenediaminetetraacetic acid that were kept cold until they arrived to the laboratory of the National Genetic Resources Center. In the laboratory, mRNA was obtained from whole blood using the commercial method SV Total RNA Isolation System (Promega, Madison, WI, USA). Later, mRNA concentration and purity were determined by spectrophotometry in a NanoDrop 2000 equipment (Thermo Fisher Scientific, Waltham, MA, USA). The synthesis of cDNA was carried out by reverse-transcribed of mRNA using GoScriptTM Reverse Transcription System (Promega, USA).

Gene expression analysis were carried out on *HSP60*, *HSP70*, and *HSP90* genes using the relative quantification by ΔΔCt comparative method. Constitutive *β*-*actin* was included as a reference gene. Target genes were amplified as duplex quantitative polymerase chain reaction (qPCR) in a StepOnePlus Real-Time System (Thermo Fisher Scientific, USA) with primers and marked probes shown in Table 1. The probes of target genes were marked with fluorophore FAM and probe of *β*-*actina* was marked with fluorophore HEX, while, quencher used in all probes was TAMRA.

Final volume of PCR reaction mix was 15 μL, which included 1X of TaqMan Fast Advanced Master Mix (Thermo Fisher Scientific, USA), 0.4 μM of each primer, 0.2 μM of probe and 0.2 μg of DNA. For the *HSP60* gene, the amplification conditions were 95°C for 10 min for pre-denaturation and 45 cycles at 95°C for 15 s, followed by 65°C for 1 min, while, for the *HSP70* and *HSP90* genes were 95°C for 10 min for pre-denaturation and 45 cycles at 95°C for 15 s, followed by 65 and 62°C, respectively for 40 s and 72°C for 40 s (Table 1).

### Statistical analysis

Expression of *HSP60*, *HSP70*, and *HSP90* genes. The PROC MIXED procedure was used in a repeated measures analysis [15], considering that two samples were taken each day from the same animal (sampling hour). Hence, the mixed model approach to the analysis of repeated measures was used to model the appropriate covariance structure of data. Criteria for selecting the adequate covariance structure were Akaike [16] and Schwarz [17]. Factors included in the model were year (2018, 2019), farm (three locations), sex (males, females), sampling hour (am, pm), age of animals as a covariate, and the interaction of sampling hour with year, farm and sex. Comparison of means was carried out by lsmeans test based on the generalized least-squares method. The statistical analysis included animals with complete relative quantification in which the gene amplification was obtained for the *HSP* gene and the reference gene *β-actin*, and with Ct values lower than 40, otherwise data was discarded.

Physiological data. Body temperature (BT°), heart rate (HR), and respiratory rate (RR) were measured twice a day in each animal. Analysis of variance were carried out for BT°, HR, and RR including the fixed effects of sex and farm [15].

## RESULTS AND DISCUSSION

Expression of *HSP60*, *HSP70*, and *HSP90* genes in Simmental cattle for sampling hour, year, farm sex and the interaction sampling hour×farm are shown in Table 2. For *HSP60* gene expression differences were found only for sex (p = 0.0349), in contrast, for the gene *HSP70* differences were observed for sampling hour (p = 0.0042), farm (p<0.0001), sex (p = 0.0476) and the interaction sampling hour×farm (p = 0.0002). For the gene *HSP90*, differences of expression were observed for farm (p<0.0001) and year (p = 0.0521) (Table 2).

In the analysis of sampling hour, between early morning (0.388±0.083) and noon (0.608±0.064), a significant difference (p = 0.0042) was observed for the *HSP70* gene expression which was lower in the morning than noon (Table 3). By contrast, Bretanha et al [5] reported in Simmental and Angus breeds that the expression of the *HSF1* and *HSP70* genes was similar in animals exposed to heat stress and control, in different sampling hours. In *HSP60* and *HSP90*, results found in this report are comparable to the obtained in Nerole and Caracu breeds, which, the expression of *HSP60* was similar during morning and afternoon in animals exposed in sun and shade [18].

Despite of previous reports *in vitro* [8,12,13,19–21] and *in vivo* [9–11,20], about increasing the *HSP* gene expression in some breeds of bovines and buffalo during heat stress conditions, in this study differences (p = 0.0042) were observed only in *HSP70* gene expression between early morning and noon (Table 3). Which is related to more comfortable environmental conditions in the early morning than noon, with the temperature increasing during afternoon [5,18]. Similar expression of *HSP* genes in both, early morning, and noon, could be due to time spent by animals acclimatizing and recovering. This was reported in fibroblasts of buffalo, which, expression of *HSP40*, *HSP60*, *HSP70*, and *HSP90* increased after 24 hours then, in bovine basal levels were recovered in eight hours [12].

Interactions of sampling hour with farm for *HSP70* gene expression are shown in Table 2. Differences in *HSP70* were observed in the interaction sampling hour×farm (p = 0.0002), which, could be due to variations in time of exposure to the sun, temperature and RH. Pires et al [18] mentioned that exposure of bovine Nerole and Caracu breeds do not affect *HSP60*, *HSP70*, and *HSP90* gene expression however, a rise of RH increases *HSP70* expression. *HSP* gene expression is widely diverse in natural environment conditions, typical of locality studied [10].

In the case of *HSP60* and *HSP90*, differences in interaction sampling hour×farm were not observed (p>0.05). This could be due to that *HSP70* gene may be better marker of heat stress in bovine than *HSP90* [9] and it is more influenced in some breeds [10]. Also, Hooper et al [19] mentioned that *HSP90* gene should not be considered as a marker for heat stress due to the low stability of expression in some temperature conditions.

Estimation of least-square means of *HSP60*, *HSP70*, and *HSP90* genes are shown in Table 3. The *HSP60* gene expression was higher in males than females. The *HSP70* gene expression was higher in: i) noon (0.608±0.064) than early morning (0.388±0.083), ii) Compostela (0.947±0.141), followed by Tangancícuaro (0.340±0.059) and Puerto Vallarta (0.208±0.056), and iii) males (0.597±0.102) than females (0.399±0.050). Gene expression at noon in Compostela was different to others sampling hour×farm interactions (Table 3). In the case of *HSP90*, gene expression in Puerto Vallarta was lower than Compostela and Tangancícuaro (Table 3).

The differences among farms found in this study for the *HSP70* gene expression could be due to natural environment conditions, which are typical of the farm, and to individual adaptation of animals to heat stress [8,10,18]. The different levels of expression can be useful to identify animals for their incorporation in genetic improvement related to tolerance to heat stress in Simmental breed [5].This issue is important considering that in tropical, subtropical and arid areas, heat stress is the main factor in reduction of livestock production [11]. Other authors have also reported about mRNA of *HSP* genes results in cattle (*Bos indicus*) and riverine buffaloes (*Bubalus bubalis*) [10–12].

On the other hand, differences on *HSP60* and *HSP70* genes were found for sex, however, number of females (78) and number of males (6) included in the analysis, should be considered. Studies in cows [9–11] and steers [5,18] have been reported separately, however, results in cattle about *HSP70* mRNA expression in both, male and female, have not been reported until now.

**Figure 1:**
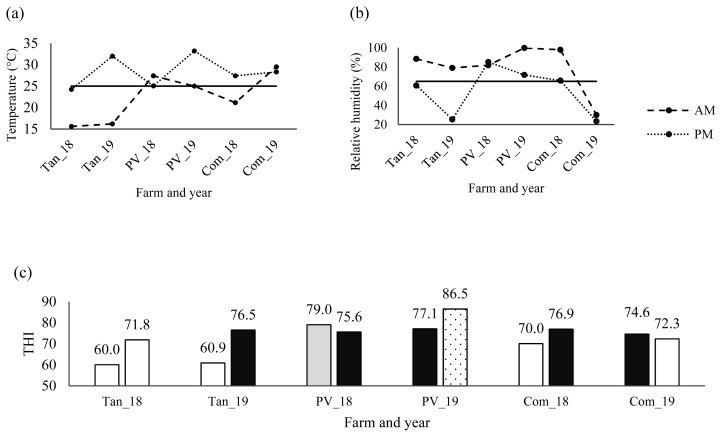
(a) Environmental temperature and (b) relative humidity of three sampled farms. Dashed lines depict to measurement in early morning; dotted lines depict to measurement in noon; solid line is temperature of 25°C in (a) and relative humidity of 65% in (b), respectively. (c) Temperature-humidity Index from three farms in 2018 and 2019, during early morning and noon. White bars show comfort conditions; black bars show heat stress alert; gray bar shows dangerous; dotted bar shows emergency. The first bar in each farm is THI value in early morning and the second bar is THI value at noon. Tan_18 = Tangancícuaro, 2018; Tan_19 = Tangancícuaro, 2019; PV_18 = Puerto Vallarta, 2018; PV_19 = Puerto Vallarta, 2019; Com_18 = Compostela, 2018; Com_19 = Compostela, 2019.

Environmental temperature and RH from farms monitored with weather stations during sampling are shown in Figures 1a and 1b. In early morning, temperatures were lower than noon at all farms except Puerto Vallarta in 2018 and Compostela in 2019, while, RH decreased at noon (Figures 1a and 1b). Differential expression of *HSP60* and *HSP90* genes were not significant for sampling hour (p = 0.8220, and p = 0.9848, respectively), in accordance with other trials related to time of sampling [5,18]. Despite the temperature below 25°C and RH above 65% in early morning (except Puerto Vallarta in 2018 and Compostela in 2019), differences among farms (p<0.0001) were found for expression of *HSP70* and *HSP90* genes (Table 2), which could be due to natural environment of temperature and RH.

On the other hand, exposure of animal to heat stress is measured by THI, with values of THI below 74 indicating thermal comfort, values from 74 to 79 indicating heat alert, values from 79 to 84 indicating a dangerous situation and values above 84 indicating emergency [22]. Values of THI in farms from western Mexico are shown in Figure 1c. In early morning, comfortable weather was observed in all farms, except in Puerto Vallarta in 2018 and Compostela in 2019 (THI = 79.0 and 74.6, respectively). At noon, two comfortable, three heat stress alert and one emergency conditions were observed (Figure 1c). These results were similar to previous report [19], of comfortable environment during morning and heat alert or emergency in afternoon, based on THI values.

Monitoring activities of environmental conditions is recommendable for areas with high THI values, as Puerto Vallarta, that induce heat stress in animals [9] which decreases productivity of livestock [11], despite of adaptability to tropical weather and the efficiency of breeding by selection [18].

Estimation of least-squares means for farm and sex related to BT°, HR, and RR are shown in Table 4. Results indicated statistical differences for farm and sex (p<0.05), with males showing a higher BT° and RR than females (39.18±0.06 and 45.95±1.63 vs 38.89±0.02 and 42.35±0.63, respectively). In agreement with this result, other studies have indicated that respiratory rate is the best physiological indicator of thermal stress in *Bos taurus* cattle [3,4]. On the other hand, results related to farm showed that, among the three sampled farms, Puerto Vallarta had the highest averages (p<0.05) for BT° and RR, which points out Puerto Vallarta as the place having the hottest weather, and the farm with animals under a great heat stress.

## CONCLUSION

Expression of *HSP70* gene in Simmental herds of the tropical region of western México was different during early morning and noon, but the expression of the *HSP60* and *HSP90* genes was similar. *HSP70* gene showed to be a better marker of heat stress than *HSP60* and *HSP90* genes. Individual adaptability of the animals on exposure to heat stress could be influenced by *HSP* gene expression. Identification of resilient animals to heat stress would allow their incorporation in genetic improvement programs for the Simmental breed.

## Figures and Tables

**Table 1:** Description of primers and probes used in the amplification of *HSP60*, *HSP70*, and *HSP90* genes in Simmental cattle

Gene	Sequence of oligo (5′ - 3′)	Amplicon size (pb)	Alignment temperature (°C)
*HSP60*	F- GCGCTAAACTTGTTCAAGATGTTGC R- CTAACATCACACCTCTCCTGATTTC P- CGGGGGATGGCACCACTACTGCT	159	65
*HSP70*	F- CGAAAAACATGGCTATCGGCATC R- CTACGTGGCCTTCACCGATAC P- GTTCCAGCACGGCAAGGTGGAGATC	135	62
*HSP90*	F- TGCAGGAGGGTCCTTCACAG R- CTCCAGGTACTCAGTCTGGTC P- CACAGGAGAACCAATGGGACGTGGAA	102	65
*β*-*actin*	F- ACTCGTACGTGGGGGATGAG R- TCCATGTCGTCCCAGTTGGTG P- GAGAGGCATCCTGACCCTCAAGTAC	94	62, 65

*HSP*, heat shock proteins; F, forward primer; R, reverse primer; P, probe.

**Table 2:** p-values for the expression of *HSP* genes for Simmental cattle in three farms of the tropical region of western México

Factor	*HSP60*	*HSP70*	*HSP90*
A: sampling hour	0.8220	0.0042	0.9848
B: year	0.1526	0.2955	0.0521
C: farm	0.2676	<0.0001	<0.0001
D: sex	0.0349	0.0476	0.3626
Interaction A×C	-	0.0002	-
Covariable: age	-	0.0651	-

*HSP*, heat shock proteins.

**Table 3:** Least-squares means and standard errors for the expression of *HSP* genes for Simmental cattle in three farms of the tropical region of western México

Factor	Level	*HSP60*	*HSP70*	*HSP90*
A: sampling hour	am	2.122±0.287^a^	0.388±0.083^a^	3.377±0.802^a^
	pm	2.067±0.287^a^	0.608±0.064^b^	3.392±0.802^a^
B: year	2018	1.807±0.253^a^	0.528±0.064^a^	4.651±0.549^a^
	2019	2.381±0.387^a^	0.468±0.075^a^	2.117±1.225^b^
C: farm	Compostela	1.915±0.417^a^	0.947±0.141^a^	5.138±0.984^a^
	Puerto Vallarta	1.850±0.352^a^	0.208±0.056^b^	0.116±0.857^b^
	Tangancícuaro	2.518±0.331^a^	0.340±0.059^c^	4.899±0.903^a^
D: sex	Female	1.553±0.219^a^	0.399±0.050^a^	2.891±0.663^a^
	Male	2.636±0.464^b^	0.597±0.102^b^	3.878±1.060^a^
Interaction A×C	am×Compostela	-	0.531±0.204^ab^	-
	pm×Compostela	-	1.363±0.141^c^	-
	am×Puerto Vallarta	-	0.258±0.074^ab^	-
	pm×Puerto Vallarta	-	0.157±0.056^a^	-
	am×Tangancícuaro	-	0.377±0.069^b^	-
	pm×Tangancícuaro	-	0.304±0.059^b^	-

*HSP*, heat shock proteins.

a–c Different letter superscripts in the same column indicate significant differences (p≤0.05).

**Table 4:** Least-squares means and standard errors for farm and sex considering body temperature (BT°), heart rate (HR) and respiratory rate (RR) of Simmental cattle in the tropical region of western México

Items	BT°	HR	RR
Farm			
Tangancícuaro	38.83±0.092^a^	92.73±3.24	34.43±2.39^a^
Compostela	39.00±0.091^ab^	93.59±3.21	39.02±2.36^b^
Puerto Vallarta	39.32±0.080^b^	99.25±2.80	69.09±2.06^c^
Sex			
Males	39.18±0.06^a^	96.85±2.20	45.95±1.63^a^
Females	38.89±0.02^b^	94.90±0.86	42.35±0.63^b^

a–c Different letter superscripts in the same column indicate significant differences (p≤0.05).
